# Knowledge, attitudes, and practice related to tooth loss and dentures among patients with dental arch deficiencies

**DOI:** 10.1186/s12889-024-19310-2

**Published:** 2024-07-06

**Authors:** Jing Sun, Junru Meng, Jianliang Shan, Huijun Lu, Wei Wei, Shengnan Zhang, Li Zhang

**Affiliations:** 1 Department of Periodontology, Jinan Key Medical and Health Laboratory of Oral Diseases and Tissue Regeneration, Jinan Key Laboratory of Oral Diseases and Tissue Regeneration, Shandong Provincial Key Medical and Health Laboratory of Oral Diseases and Tissue Regeneration, Shandong Provincial Key Medical and Health Discipline of Oral Medicine, Jinan Stomatological Hospital, Jinan, Shandong 250001 China; 2 Hospital Infection Management Office, Jinan Key Medical and Health Laboratory of Oral Diseases and Tissue Regeneration, Jinan Key Laboratory of Oral Diseases and Tissue Regeneration, Shandong Provincial Key Medical and Health Laboratory of Oral Diseases and Tissue Regeneration, Shandong Provincial Key Medical and Health Discipline of Oral Medicine, Jinan Stomatological Hospital, Jinan, Shandong 250001 China; 3 Department of Prosthodontics, Jinan Key Medical and Health Laboratory of Oral Diseases and Tissue Regeneration, Jinan Key Laboratory of Oral Diseases and Tissue Regeneration, Shandong Provincial Key Medical and Health Laboratory of Oral Diseases and Tissue Regeneration, Shandong Provincial Key Medical and Health Discipline of Oral Medicine, Jinan Stomatologic Hospital Shungeng Branch, Jinan, Shandong 250001 China; 4 Department of Prosthodontics, Jinan Key Medical and Health Laboratory of Oral Diseases and Tissue Regeneration, Jinan Key Laboratory of Oral Diseases and Tissue Regeneration, Shandong Provincial Key Medical and Health Laboratory of Oral Diseases and Tissue Regeneration, Shandong Provincial Key Medical and Health Discipline of Oral Medicine, Jinan Stomatological Hospital East Branch, Jinan Stomatological Hospital, No.52, Huanshan Road, Lixia District, Jinan, Shandong 250014 China; 5 Department of Prosthodontics, Beijing Stomatological Hospital, School of Stomatology, Capital Medical University, Beijing, 100000 China; 6 Cosmetic Dentistry, Jinan Key Medical and Health Laboratory of Oral Diseases and Tissue Regeneration, Jinan Key Laboratory of Oral Diseases and Tissue Regeneration, Shandong Provincial Key Medical and Health Laboratory of Oral Diseases and Tissue Regeneration, Shandong Provincial Key Medical and Health Discipline of Oral Medicine, Jinan Stomatological Hospital, No.82, Wei’er Road, Shizhong District, Jinan, Shandong 250001 China

**Keywords:** Tooth loss, Dentures, Knowledge, Attitude, Practice, Dental arch deficiencies

## Abstract

**Background:**

Tooth loss is a common problem that affects many people worldwide. Exploring knowledge, attitude, and practice (KAP) among patients can identify barriers and challenges in following recommended practices, providing valuable insights for dental healthcare providers, policymakers, and researchers. This study aimed to explore the KAP of patients with dental arch deficiencies regarding tooth loss and dentures.

**Methods:**

This web-based, cross-sectional study was conducted among patients with dental arch deficiencies using a self-designed questionnaire.

**Result:**

3166 valid questionnaires were included. Participants’ mean KAP scores were 6.84 ± 2.27 (possible range: 0 ~ 12), 39.4 ± 3.72 (possible range: 9 ~ 45), and 27.7 ± 4.36 (possible range: 8 ~ 40), respectively. Multivariable logistic regression analysis showed that knowledge (OR = 1.383), employed (OR = 1.805), family history (OR = 2.158), and treatment (OR = 1.683) were independently associated with attitude. Moreover, knowledge (OR = 1.239), attitude (OR = 1.250), female (OR = 0.619), age (OR = 0.967), college/bachelor (OR = 0.373), and master and above degree (OR = 0.418), employed (OR = 0.554) or student (OR = 0.434), with 10,001–20,000 Yuan household income per month (OR = 0.492), have been married (OR = 0.609), smoking (OR = 0.595), drinking (OR = 0.397), disease duration (OR = 0.972), with family history (OR = 1.676), and with treatment (OR = 3.492) were independently associated with practice (all *P* < 0.05).

**Conclusion:**

Patients with dental arch deficiencies have insufficient knowledge, positive attitudes, and moderate practice toward tooth loss and dentures, which might be affected by multiple demographic factors.

**Supplementary Information:**

The online version contains supplementary material available at 10.1186/s12889-024-19310-2.

## Background

Tooth loss is a common problem that affects many people worldwide [1], and 2.4% of the population suffers from severe tooth loss [2], while the 2030 predicted prevalence of tooth loss in younger adults and younger seniors would be 50% and 65%, respectively [3]. Tooth loss can be caused by dental decay, gum disease, accidents, congenital conditions, or aging [4]. Tooth loss can cause discomfort, impair oral functions (eating and speaking), and affect oral health (occlusion issues, tooth displacement, and open wounds), self-confidence, and overall well-being [5]. The options available for tooth loss include dentures and dental implants. Dental implants are expensive and involve a highly invasive procedure with complications like jaw fracture, but they are long-lasting and effective [6]. Dentures provide a reliable solution for individuals seeking to replace missing teeth [7]. Dentures, either partial or complete, are custom-made based on an initial assessment of oral health, and these are crafted to fit comfortably and resemble natural teeth [8]. Regular maintenance and follow-up are important for denture longevity and to avoid carries and sensitivity to the adjacent teeth. While dentures are cost-effective, dental implants may be suggested as a more permanent option. Tooth loss and dentures are crucial aspects of dental care. Good knowledge about tooth loss and dentures indicates awareness of causes, consequences, treatment options, and the benefits and limitations of dentures [9].

Knowledge, attitude, and practice (KAP) studies can help identify the gaps, misconceptions, and misunderstandings regarding a particular health issue. This study could help identify areas for further education and information. Indeed, the attitude of patients towards tooth loss and dentures significantly affects their decision-making and well-being [10]. It uncovers the emotional and psychological aspects of their experiences, including self-esteem, body image, and social interactions [11]. A positive attitude promotes the willingness to seek treatment and adhere to oral care routines, potentially improving their quality of life [12]. Understanding the practices and behaviors of patients with dental arch deficiencies is crucial for evaluating the efficacy of dentures [13]. Exploring KAP among patients can identify barriers and challenges in following recommended practices, providing valuable insights for dental healthcare providers, policymakers, and researchers. Older adults know that they can replace lost teeth with dentures, but they are unaware of the importance of oral care [14]. Although dental practitioners have a good knowledge of prosthodontics [15], as could be expected, the patients’ KAP regarding tooth loss and dentures is mostly unknown.

This study investigated the KAP of patients regarding tooth loss and dentures in dental arch deficiencies. The hypothesis was that individuals have moderate KAP toward dentures, that specific socioeconomic factors influence the KAP, and that better knowledge translates into more positive attitudes and more proactive practice.

## Methods

### Study design and participants

This cross-sectional investigation was conducted at Jinan Stomatological Hospital between January and May 2023. The study enrolled patients with dental arch deficiencies, included patients aged 16 to 80 seeking dental care, and excluded infants, toddlers, and pregnant women. This study was approved by the Ethics Committee of Jinan Stomatological Hospital (JNSKQYY-2023-003). Informed consent was obtained from all participants.

### Questionnaire

The questionnaire was designed by the investigators and was in Chinese. The initial questionnaire design was reviewed by three experts, and its reliability was evaluated, which showed a Cronbach’s α coefficient of 0.861, indicating excellent internal consistency. The final questionnaire was in Chinese and consisted of four dimensions for data collection, totaling 41 items. These dimensions were basic information, knowledge, attitude, and practice, having 15, 12, 10, and 8 items, respectively. Within the knowledge dimension, each item had correct and incorrect answers, receiving 1 point and 0 points, respectively. The cutoff value for the knowledge dimension was set at 70%, with < 8 points indicating insufficient knowledge. The attitude and practice dimensions primarily used a five-point Likert scale, ranging from ‘very positive’ (5 points) to ‘very negative’ (1 point). For items 1–9 in the attitude dimension, the options ‘strongly agree’, ‘agree’, ‘neutral’, ‘disagree’, and ‘strongly disagree’ correspond to 5, 4, 3, 2, and 1 points, respectively. However, for items 7 and 10, the scoring was reversed. The cutoff value for the attitude dimension was set at 9–22 for negative attitude, 23–32 for moderate attitude, and 33–45 for positive attitude. In the practice dimension, items 1 to 7 were scored with options ‘strongly agree’, ‘agree’, ‘neutral’, ‘disagree’, and ‘strongly disagree’ corresponding to 5, 4, 3, 2, and 1 point, respectively. However, for item 8, the scoring was reversed. The cutoff value for practice dimension was set at 8–20 for inactive practice, 21–29 for moderate practice, and 30–40 for proactive practice.

Convenient sampling was used to recruit patients for outpatient care, and the research assistant questioned and assessed whether the patients met the requirements. Six trained research assistants participated in the distribution and collection of questionnaires. Paper questionnaires and online questionnaires (using the Questionnaire Star app) were completed in the outpatient clinic by the study assistant. Before filling in the questionnaire, the respondents were asked about their wishes, and after the wishes were filled in, they were instructed to fill in the questionnaire requirements. They were required to fill in the questionnaire truthfully. If they did not understand, the research assistant would help them carefully explain. After completing 50 questionnaires, the research assistant checked the completion of the questionnaire and obvious logic problems and sought professional statisticians to conduct questionnaire quality control, including response time, logic, obvious errors, etc. After passing the assessment, large-scale delivery and questionnaire collection began similarly. Incomplete questionnaires, questionnaires with obvious logic errors (e.g., impossible age), or those filled using all the same options (e.g., all first options) were excluded. Only one questionnaire could be submitted using a given IP address for the online questionnaires.

### Sample size

The sample size was determined based on the formula for cross-sectional surveys:

$$n={\left(\frac{{Z}_{1}-\alpha /2}{\delta }\right)}^{2}\times p\times (1-p)$$

In the formula, *n* represents the sample size for each group, *α* represents the type I error (which is typically set at 0.05), *Z*_*1−α/2*_=1.96, *δ* represents the allowable error (typically set at 0.05), and *p* is set at 0.5 (as setting it at 0.5 maximizes the value and ensures a sufficiently large sample size). Hence, the calculated sample size was 384. Considering an estimated questionnaire response rate of 80%, 480 valid questionnaires were needed. In order to improve representativeness and generalizability, we have significantly increased the sample size where we can. Finally, this study enrolled 3166 participants.

### Statistical analysis

Stata 17.0 (Stata Corporation, College Station, TX, USA) software was used for statistical analysis. Data for different demographic characteristics and responses to individual questions were counted as n (%). Continuous variables were described using mean ± SD, t-test, or analysis of variance were used to compare different groups. Pearson’s correlation analysis was used to examine correlations among the three dimensions. Multivariable logistic regression analysis was conducted to explore the associations between KAP and demographic information. A two-tailed P-value of < 0.05 was considered statistically significant.

## Results

The questionnaire was distributed among 3166 patients with dental arch deficiencies, and all responded. The mean knowledge, attitude, and practice scores were 6.84 ± 2.27 (possible range: 0 ~ 12), 39.4 ± 3.72 (possible range: 9 ~ 45), and 27.7 ± 4.36 (possible range: 8 ~ 40), respectively. The results showed that males had higher scores than females. Participants from rural areas had the highest scores, followed by those from urban and suburban areas, and highly educated patients had better scores than less educated patients. Similarly, employed individuals had the highest scores, and those with higher household incomes had higher scores. Unmarried, divorced, or widowed participants had higher scores than married individuals. Participants who smoked or drank had higher scores, as did those with social medical insurance and those with a family history of dental arch deficiencies, underlying medical conditions, or who received treatment (all *P* < 0.05) (Table 1).

**Table 1:** Demographic information of patients with dental arch deficiencies

Variables	*N* (%)	Knowledge		Attitude		Practice	
		Mean ± SD	*P*-value	Mean ± SD	*P*-value	Mean ± SD	*P*-value
**Total**	3166 (100)	6.84±2.27		39.4±3.72		27.7±4.36	
**Gender**			**<0.001**		**<0.001**		**<0.001**
Male	1129(35.66)	8.21±1.48		41.65±2.73		30.81±3.8	
Female	2037(64.34)	6.08±2.27		38.15±3.61		25.97±3.64	
**Age**	52.26±14.22						
**Residence**			**<0.001**		**<0.001**		**<0.001**
Rural	111(3.51)	8.33±1.56		42.02±3.36		31.51±3.7	
Urban	2485(78.49)	7.14±2.18		39.89±3.57		28.25±4.32	
Suburban	570(18)	5.27±2.02		36.73±3.12		24.52±2.74	
**Education**			**<0.001**		**<0.001**		**<0.001**
≤ Middle School	240(7.58)	8.32±1.29		42.57±1.91		31.89±3.62	
High School	358(11.31)	8.35±1.25		42.31±2.12		31.53±3.5	
Bachelor	2186(69.05)	6.72±2.29		39.01±3.59		27.1±3.99	
Master and above	382(12.07)	5.22±2		36.88±3.64		24.88±3.46	
**Occupation**			**<0.001**		**<0.001**		**<0.001**
Employed	1734(54.77)	7.81±1.8		40.98±3.1		29.72±4.09	
Unemployed	895(28.27)	5.68±2.22		37.45±3.48		25.29±3.24	
Self-employed	273(8.62)	5.69±2.25		37.46±3.68		25.37±3.57	
Student	264(8.34)	5.6±2.17		37.62±3.46		24.98±3.21	
**Household income, Yuan per month**			**<0.001**		**<0.001**		**<0.001**
<5,000	236(7.45)	8.32±1.29		42.46±2.22		31.69±3.91	
5,001-10,000	1071(33.83)	8.07±1.61		41.3±2.88		30.19±3.86	
1,0001-20,000	623(19.68)	6.75±2.3		39.17±3.57		27.13±3.92	
>20,000	74(2.34)	6.2±2.24		38.57±3.27		26.51±3.75	
Prefer not to say	1162(36.7)	5.5±2.12		37.2±3.39		24.96±3.04	
**Marital status**			**<0.001**		**<0.001**		**<0.001**
Unmarried/Divorced/Widowed	616(19.46)	8.15±1.73		41.73±3.06		30.79±4.24	
Married	2256(71.26)	6.73±2.24		39.15±3.62		27.3±4.09	
Prefer not to say	294(9.29)	4.96±1.84		36.4±2.92		24.22±2.42	
**Smoking**			**<0.001**		**<0.001**		**<0.001**
Yes	465(14.69)	6.57±2.29		38.89±3.65		26.98±4.09	
No	2701(85.31)	8.4±1.34		42.34±2.6		31.85±3.48	
**Drinking**			**<0.001**		**<0.001**		**<0.001**
Yes	575(18.16)	6.48±2.27		38.75±3.62		26.79±4.02	
No	2591(81.84)	8.48±1.3		42.34±2.61		31.76±3.46	
**Medical insurance**			**<0.001**		**<0.001**		**<0.001**
Social medical insurance	1841(58.15)	7.79±1.8		40.93±3.13		29.52±4.1	
Commercial medical insurance	235(7.42)	6.07±2.26		37.87±3.42		25.7±3.47	
Both social and commercial medical insurance	919(29.03)	5.51±2.17		37.34±3.44		25.24±3.32	
No medical insurance	171(5.4)	4.87±2.03		36.12±3.13		24±2.97	
**Disease duration**	19.82±12.42						
**Family history**			**<0.001**		**<0.001**		**<0.001**
Yes	1616(51.04)	7.93±1.76		41.15±3		29.96±4.02	
No	889(28.08)	6.13±2.27		38.18±3.62		25.99±3.62	
Not sure	661(20.88)	5.15±1.93		36.74±3.19		24.46±2.67	
**With treatment**			**<0.001**		**<0.001**		**<0.001**
Yes	2661(84.05)	6.94±2.22		39.52±3.6		27.96±4.28	
No	505(15.95)	6.3±2.42		38.76±4.27		26.31±4.55	
**Underlying diseases**			**<0.001**		**<0.001**		**<0.001**
Yes	2036(64.31)	6.56±2.3		38.95±3.67		27.19±4.16	
No	1130(35.69)	7.36±2.12		40.2±3.69		28.6±4.57	

The analysis of the knowledge dimension showed that the highest percentage of correct answers (85.72%) was recorded for question K1, which asked about understanding dental arch deficiency and its impact on the total number of teeth. On the other hand, question K7, which asked about the suitability of fixed dental prostheses for cases with a higher number of missing teeth and healthy adjacent teeth and periodontal tissues, received the lowest percentage of correct answers (2.97%). The highest rate of incorrect answers (97.03%) was also for question K7, while question K1 had the lowest percentage of wrong answers (14.28%) (Table S1).

The distribution of attitude dimension revealed a wide spectrum of responses to the ‘strongly agree’ option among participants. Question A3, concerning the effect of missing teeth on teeth alignment and its consequences, received the highest agreement rate at 54.17%. In contrast, question A4, which states that missing teeth can lead to other oral conditions such as periodontal disease and dental caries, had the lowest agreement rate at 22.3% (Table S2).

Regarding practice attitudes, participants responded to the ‘always’ option in various ways. The highest percentage (27.8%) was recorded for question P7, which indicated a willingness to follow medical advice and cooperate with all pretreatment checks and treatments before dentures. The lowest percentage (6.31%) was for question P8, indicating an unwillingness to use dentures to treat missing teeth (Table S3).

Pearson’s correlation analysis showed a positive correlation between knowledge and attitude (*r* = 0.646, *P* < 0.001), between knowledge and practice (*r* = 0.661, *P* < 0.001), and between attitude and practice (*r* = 0.705, *P* < 0.001) (Table 2). Furthermore, multivariable logistic regression analysis showed that female (OR = 0.561, 95% CI: 0.445–0.707, *P* < 0.001), age (OR = 0.956, 95% CI: 0.947–0.966, *P* < 0.001), college/bachelor (OR = 0.572, 95% CI: 0.354–0.924, *P* = 0.022) and master or above degree (OR = 0.378, 95% CI: 0.212–0.674, *P* = 0.001), employed (OR = 1.655, 95% CI: 1.287–2.127, *P* < 0.001), have been married (OR = 0.618, 95% CI: 0.455–0.84, *P* = 0.002), drinking (OR = 0.65, 95% CI: 0.478–0.811, *P* = 0.004), with social medical insurance (OR = 2.821, 95% CI: 1.59–5.005, *P* < 0.001), disease duration (OR = 0.989, 95% CI: 0.98–0.999, *P* = 0.035), with family history (OR = 1.821, 95% CI: 1.443–2.298, *P* < 0.001), with treatment (OR = 2.332, 95% CI: 1.68–3.237, *P* < 0.001), with underlying diseases (OR = 0.745, 95% CI: 0.597–0.929, *P* = 0.009) were independently associated with knowledge (Table 3). Knowledge (OR = 1.383, 95% CI: 1.235–1.55, *P* < 0.001), employed (OR = 1.805, 95% CI: 1.026–3.176, *P* = 0.040), family history (OR = 2.158, 95% CI: 1.121–4.152, 95% CI: 0.021), and with treatment (OR = 1.683, 95% CI: 1.088–2.603, *P* = 0.019) were independently associated with attitude (Table 4). Moreover, knowledge (OR = 1.239, 95% CI: 1.141–1.345, *P* < 0.001), attitude (OR = 1.25, 95% CI: 1.187–1.318, *P* < 0.001), female (OR = 0.619, 95% CI: 0.484–0.792, *P* < 0.001), age (OR = 0.967, 95% CI: 0.956–0.978, *P* < 0.001), college/bachelor (OR = 0.373, 95% CI: 0.237–0.587, *P* < 0.001) and master and above degree (OR = 0.418, 95% CI: 0.211–0.828, *P* = 0.012), employed (OR = 0.554, 95% CI: 0.401–0.766, *P* < 0.001) or student (OR = 0.434, 95% CI: 0.211–0.828, *P* = 0.033), with 10,001–20,000 Yuan household income per month (OR = 0.492, 95% CI: 0.307–0.79, *P* = 0.003), have been married (OR = 0.609, 95% CI: 0.45–0.824, *P* < 0.001), smoking (OR = 0.595, 95% CI: 0.471–0.812, *P* = 0.006), drinking (OR = 0.397, 95% CI: 0.251–0.585, *P* = 0.029), disease duration (OR = 0.972, 95% CI: 0.96–0.984, *P* < 0.001), with family history (OR = 1.676, 95% CI: 1.257–2.234, *P* < 0.001), and with treatment (OR = 3.492, 95% CI: 2.307–5.285, *P* < 0.001) were independently associated with practice (Table 5).

**Table 2:** Pearson’s correlation analysis

	Knowledge	Attitude	Practice
**Knowledge**	-		
**Attitude**	0.646 (P**<**0.001)	-	
**Practice**	0.661 (P**<**0.001)	0.705 (P**<**0.001)	-

**Table 3:** Multivariable logistic regression analysis for knowledge dimension about tooth loss and dentures

Knowledge	Univariable logistic regression		Multivariable logistic regression	
	OR (95% CI)	*P*-value	OR (95% CI)	*P*-value
**Gender**				
Male	REF		REF	
Female	0.112(0.094-0.133)	<0.001	0.561(0.445-0.707)	<0.001
**Age**	0.904(0.897-0.911)	<0.001	0.956(0.947-0.966)	<0.001
**Residence**				
Rural	REF		REF	
Urban	0.124(0.066-0.233)	<0.001	0.793(0.374-1.679)	0.544
Suburban	0.019(0.01-0.037)	<0.001	0.453(0.204-1.006)	0.052
**Education**				
≤ Middle School	REF		REF	
High School	0.95(0.596-1.513)	0.828	1.009(0.58-1.754)	0.975
College/Bachelor	0.123(0.085-0.179)	<0.001	0.572(0.354-0.924)	0.022
Master and above	0.029(0.018-0.046)	<0.001	0.378(0.212-0.674)	0.001
**Occupation**				
Unemployed	REF		REF	
Employed	7.143(5.933-8.599)	<0.001	1.655(1.287-2.127)	<0.001
Self-employed	1.066(0.776-1.465)	0.692	1.143(0.763-1.713)	0.516
Student	0.781(0.553-1.103)	0.161	0.861(0.561-1.322)	0.495
**Household income, Yuan per month**				
<5,000	REF		REF	
5,001-10,000	0.478(0.324-0.704)	<0.001	1.203(0.757-1.913)	0.434
10,001-20,000	0.13(0.087-0.193)	<0.001	0.882(0.542-1.437)	0.615
>20,000	0.067(0.036-0.124)	<0.001	0.671(0.313-1.437)	0.304
Prefer not to say	0.039(0.026-0.057)	<0.001	0.58(0.357-0.945)	0.029
**Marital Status**				
Unmarried/Divorced/Widowed	REF		REF	
Married	0.193(0.156-0.24)	<0.001	0.618(0.455-0.84)	0.002
Prefer not to say	0.023(0.015-0.037)	<0.001	0.283(0.165-0.484)	<0.001
**Smoking**				
No	REF		REF	
Yes	0.215(0.159-0.460)	<0.001	0.25(0.868-1.8)	0.230
**Drinking**				
No	REF		REF	
Yes	0.381(0.162-0.519)	<0.001	0.65(0.478-0.811)	0.004
**Medical insurance**				
No medical insurance	REF		REF	
Social medical insurance	14.958(9.377-23.861)	<0.001	2.821(1.59-5.005)	<0.001
Commercial medical insurance	2.849(1.664-4.875)	<0.001	1.613(0.835-3.117)	0.155
Both social and commercial medical insurance	1.610(0.990-2.617)	0.055	1.238(0.686-2.233)	0.479
**Disease duration**	0.946(0.94-0.952)	<0.001	0.989(0.98-0.999)	0.035
**Family history**				
No	REF		REF	
Yes	5.722(4.784-6.845)	<0.001	1.821(1.443-2.298)	<0.001
Not sure	0.326(0.249-0.428)	<0.001	0.523(0.378-0.722)	<0.001
**With treatment**				
No	REF		REF	
Yes	1.732(1.423-2.109)	<0.001	2.332(1.68-3.237)	<0.001
**Underlying diseases**				
No	REF		REF	
Yes	0.506(0.437-0.586)	<0.001	0.745(0.597-0.929)	0.009

**Table 4:** Multivariable logistic regression analysis for attitude dimension about tooth loss and dentures

Attitude	Univariable logistic regression		Multivariable logistic regression	
	OR (95% CI)	*P*-value	OR (95% CI)	*P*-value
Knowledge	1.664(1.522-1.819)	<0.001	1.383(1.234-1.549)	<0.001
**Gender**				
Male	REF		REF	
Female	0.208(0.116-0.372)	<0.001	0.866(0.389-1.928)	0.725
**Age**	0.948(0.933-0.964)	<0.001	0.999(0.977-1.021)	0.921
**Residence**				
Rural	REF		REF	
Urban	0.958(0.297-3.093)	0.943	3.707(0.912-15.063)	0.067
Suburban	0.302(0.092-0.988)	0.048	2.624(0.621-11.087)	0.190
**Occupation**				
Unemployed	REF		REF	
Employed	5.729(3.532-9.293)	<0.001	1.805(1.026-3.176)	0.040
Self-employed	0.974(0.578-1.641)	0.922	1.041(0.602-1.801)	0.886
Student	1.375(0.758-2.494)	0.294	1.451(0.783-2.686)	0.237
**Household income, Yuan per month**				
<5,000	REF		REF	
5,001-10,000	0.824(0.181-3.741)	0.802	0.825(0.163-4.161)	0.815
10,001-20,000	0.245(0.057-1.053)	0.059	0.613(0.119-3.173)	0.560
>20,000	0.202(0.033-1.235)	0.083	0.703(0.096-5.134)	0.728
Prefer not to say	0.11(0.027-0.449)	0.002	0.492(0.098-2.478)	0.390
**Marital Status**				
Unmarried/Divorced/Widowed	REF		REF	
Married	0.28(0.129-0.607)	0.001	0.626(0.271-1.443)	0.271
Prefer not to say	0.124(0.053-0.289)	<0.001	0.602(0.239-1.522)	0.284
**Smoking**				
Yes	2.883(1.335-6.225)	0.007	0.638(0.199-2.049)	0.450
No	REF		REF	
**Drinking**				
Yes	3.232(1.569-6.659)	0.001	0.485(0.159-1.477)	0.203
No	REF		REF	
**Medical insurance**				
Social medical insurance	6.985(3.608-13.523)	<0.001	1.554(0.743-3.249)	0.241
Commercial medical insurance	1.316(0.632-2.741)	0.463	0.761(0.351-1.648)	0.488
Both social and commercial medical insurance	1.263(0.703-2.272)	0.463	0.909(0.491-1.682)	0.762
No medical insurance	REF		REF	
**Disease duration**	0.98(0.965-0.995)	0.009	1.008(0.991-1.024)	0.360
**Family history**				
No	REF		REF	
Yes	5.207(2.921-9.284)	<0.001	2.158(1.121-4.152)	0.021
Not sure	0.512(0.343-0.765)	0.001	0.729(0.478-1.111)	0.141
**With treatment**				
Yes	2.326(1.556-3.477)	<0.001	1.683(1.088-2.603)	0.019
No	REF		REF	
**Underlying medical conditions**				
Yes	0.666(0.443-1.000)	0.050		
No	REF			

**Table 5:** Multivariable logistic regression analysis for practice dimension about tooth loss and dentures

Practice Dimension	Univariable logistic regression		Multivariable logistic regression	
	OR (95% CI)	*P*-value	OR (95% CI)	*P*-value
Knowledge	2.259(2.122-2.406)	<0.001	1.239(1.141-1.345)	<0.001
**Attitude**	1.788(1.714-1.865)	<0.001	1.25(1.187-1.318)	<0.001
**Gender**				
Male	REF		REF	
Female	0.106(0.089-0.125)	<0.001	0.619(0.484-0.792)	<0.001
**Age**	0.906(0.899-0.913)	<0.001	0.967(0.956-0.978)	<0.001
**Residence**				
Rural	REF		REF	
Urban	0.158(0.098-0.254)	<0.001	0.891(0.477-1.663)	0.716
Suburban	0.01(0.006-0.019)	<0.001	0.463(0.21-1.02)	0.056
**Education**				
≤ Middle School	REF		REF	
High School	0.725(0.487-1.08)	0.114	0.746(0.447-1.245)	0.262
College/Bachelor	0.091(0.065-0.126)	<0.001	0.373(0.237-0.587)	<0.001
Master and above	0.019(0.011-0.031)	<0.001	0.418(0.211-0.828)	0.012
**Occupation**				
Unemployed	REF		REF	
Employed	9.541(7.586-11.999)	<0.001	1.805(1.305-2.496)	<0.001
Self-employed	0.846(0.537-1.334)	0.472	0.759(0.415-1.389)	0.371
Student	0.484(0.276-0.849)	0.011	0.434(0.211-0.828)	0.033
**Household income, Yuan per month**				
<5,000	REF		REF	
5,001-10,000	0.43(0.309-0.598)	<0.001	0.928(0.604-1.428)	0.735
10,001-20,000	0.098(0.069-0.14)	<0.001	0.492(0.307-0.79)	0.003
>20,000	0.068(0.035-0.13)	<0.001	0.818(0.345-1.94)	0.648
Prefer not to say	0.023(0.016-0.034)	<0.001	0.426(0.259-0.701)	0.001
**Marital Status**				
Unmarried/Divorced/Widowed	REF		REF	
Married	0.175(0.144-0.212)	<0.001	0.609(0.45-0.824)	0.001
Prefer not to say	0.011(0.005-0.023)	<0.001	0.404(0.166-0.982)	0.046
**Smoking**				
Yes	11.413(8.944-14.563)	<0.001	1.595(1.147-2.218)	0.006
No	REF		REF	
**Drinking**				
Yes	11.606(9.322-14.451)	<0.001	1.397(1.035-1.885)	0.029
No	REF		REF	
**Medical insurance**				
Social medical insurance	24.872(11.609-53.286)	<0.001	1.503(0.574-3.941)	0.407
Commercial medical insurance	3.429(1.468-8.005)	0.004	0.904(0.306-2.667)	0.854
Both social and commercial medical insurance	2.733(1.246-5.994)	0.012	1.578(0.585-4.252)	0.367
No medical insurance	REF		REF	
**Disease duration**	0.938(0.932-0.944)	<0.001	0.972(0.96-0.984)	<0.001
**Family history**				
No	REF		REF	
Yes	7.139(5.807-8.777)	<0.001	1.676(1.257-2.234)	<0.001
Not sure	0.223(0.145-0.343)	<0.001	0.610(0.358-1.038)	0.068
**With treatment**				
Yes	2.207(1.755-2.774)	<0.001	3.492(2.307-5.285)	<0.001
No	REF		REF	
**Underlying diseases**				
Yes	0.567(0.487-0.660)	<0.001	0.850(0.655-1.102)	0.220
No	REF		REF	

## Discussion

This study found that patients with dental arch deficiencies have insufficient knowledge, positive attitude, and moderate practice toward tooth loss and dentures, which might be affected by demographic factors, including gender, age, education, employment, household income, marital status, smoking, drinking, disease duration, medical insurance, family history, treatment, and underlying diseases. These findings have implications for tailoring patient education and interventions, improving access to dental care, and addressing the specific needs of individuals with dental arch deficiencies.

The analysis of demographic characteristics of patients with dental arch deficiencies revealed several important insights. Males had higher KAP scores than females, supported by previous studies that reported differences in healthcare-seeking behavior and oral health knowledge between males and females [16, 17]. Participants from rural areas had higher KAP scores than those from urban and suburban areas, which might be because people in rural areas have limited access to dental care facilities and are more cautious about oral health [18]. Highly educated and employed individuals showed better KAP scores, as supported by previous studies that reported a positive correlation between education, stable jobs, and oral health awareness and practices [19, 20]. Self-employed individuals often have higher KAP due to greater financial resources for dental care [21]. Participants with higher household incomes had higher KAP scores. In contrast, those who did not disclose their income scored lower, showing the influence of economic status on oral health, as previously reported [22]. Unmarried, divorced, or widowed participants had higher KAP scores. The literature suggests that marital status can influence healthcare behaviors [23]. Previous studies suggested that participants who smoked and drank had higher KAP scores, possibly indicating their awareness of the negative effects of these habits on oral health [24, 25]. Participants with social medical insurance had higher KAP scores than those with commercial or no insurance, emphasizing the impact of healthcare access through insurance on oral health outcomes [26]. Participants with a family history of dental arch deficiencies and those who had received treatment had higher KAP scores; those factors are known to increase the perceived importance of proactive dental care practices [27, 28]. These findings emphasize the need to tailor oral health education and interventions to specific demographic groups for better oral health outcomes. Taken together, these results suggest that the socioeconomic status appears to be a major contributor to the KAP toward missing teeth and dentures. Indeed, the socioeconomic status is associated with health literacy in general [29].

The results of the KAP dimensions showed that most patients understood their dental condition well, aligning with previous literature that suggests individuals often have a basic understanding of their dental health [30]. Still, the study identified a knowledge gap regarding the suitability of fixed dental prostheses, indicating a need for improved patient education on treatment options for missing teeth [31]. The study also revealed that patients may have misconceptions about treatment choices for dental arch deficiency, highlighting a critical area for intervention and education [32]. While most patients were aware of tooth loss’s aesthetic and functional concerns [33], some underestimated the broader oral health implications of tooth loss, emphasizing the importance of educating patients about the potential consequences [34]. Patients placed importance on suitability and comfort when making decisions about dentures, aligning with previous research that emphasizes the role of patient satisfaction in treatment success [35, 36]. Most patients were willing to follow medical advice and cooperate with pre-treatment checks and treatments, highlighting the importance of patient compliance and cooperation in achieving successful outcomes [37]. However, some patients were reluctant to choose dentures to treat missing teeth, indicating that patient preferences for treatment options vary [38]. In addition, some patients were inconsistent in oral health and denture practices, emphasizing the need for continuous patient education and support to improve oral health behaviors [39]. Hence, these findings underscore the importance of tailored patient education and interventions to bridge knowledge gaps, improve attitudes toward tooth loss consequences, and encourage consistent oral health practices among patients with dental arch deficiencies.

A study from India also showed their participants had a moderate KAP toward the replacement of missing teeth; although 83% felt the need to replace missing teeth, 57% highlighted financial limitations, and most preferred fixed partial prostheses [40]. Another study from India reported that 45% of the participants were willing to replace missing teeth, and most (72%) had a fixed partial denture [41]. Akeel [42] also reported that 82% of their participants were willing to replace missing teeth. In developing countries, the socioeconomic status and the lack of government support for dental care are major impediments to getting dentures for missing teeth [43]. In the present study, most participants indicated an unwillingness to use dentures to treat missing teeth, but the exact reasons were not explored.

Pearson’s correlation analysis revealed significant associations between KAP scores and dental arch deficiencies. Individuals with a better understanding of dental arch deficiency tended to have more positive attitudes and engaged in appropriate oral healthcare practices. It suggests that knowledge shapes attitude and drives positive oral health behaviors [44]. Similarly, the moderate positive correlation between knowledge and practice indicates that individuals with higher knowledge scores are more likely to translate their awareness into action through appropriate oral healthcare practices [45]. Moreover, individuals with positive attitudes were more inclined to adopt recommended oral healthcare practices, suggesting that a favorable attitude can be a key driver for adopting beneficial dental practices [46]. These findings highlight the importance of considering the various dimensions of KAP in the context of dental arch deficiencies.

The multivariable regression analysis provided valuable insights into the factors associated with KAP related to dental arch deficiency. The study found that various demographic and lifestyle factors were significantly associated with KAP scores. Gender was found to have a significant association with knowledge, with previous studies indicating that gender can impact oral health knowledge [47, 48]. Older individuals were found to have lower knowledge about dental arch deficiencies, which may be due to generational differences in access to oral health information [49]. Suburban residents and self-employed individuals were found to have slightly lower odds of knowledge, possibly due to differences in healthcare access and information availability [50–52]. The study also found a significant positive association between knowledge and attitude, highlighting the importance of knowledge in shaping the attitude of individuals toward dental arch deficiency [53]. Unemployed individuals were found to have lower odds of a positive attitude, reflecting the psychological and financial stress associated with unemployment [54]. Marital status was found to influence attitude, with being married associated with lower odds of a positive attitude. It may be related to differences in social support and responsibilities [55, 56]. Non-smokers and non-drinkers were found to have lower odds of a positive attitude, indicating that these behaviors influence individuals’ perspectives on oral health [57, 58]. The study highlighted the association of knowledge and attitude with oral healthcare practices [59]. Females and older individuals were found to have lower odds of positive practices, suggesting the need for targeted interventions to address gender and age-related disparities [60]. Education was found to be positively associated with practice, emphasizing the role of education in promoting healthier dental practices [24, 61–64]. Hence, this study highlights various factors influencing the KAP of individuals related to dental arch deficiency. Targeted interventions should consider these factors to enhance oral health promotion and education efforts. Targeted educational programs should address specific knowledge gaps, attitudes, or practices related to dental arch deficiencies, and collaborations between dental healthcare providers and policymakers are recommended to improve patient care and access to dental services.

This study has several limitations. First, it was conducted at a single center, and data was collected at a single point in time, limiting the applicability of the findings to other settings or populations. Establishing causality or determining the temporal relationships between variables is also impossible. Second, the study used convenient sampling and relied on self-reported responses, which may introduce selection and recall bias. The survey was designed by the investigators and can be influenced by local practices and policies, limiting the possible comparisons among studies. Third, data were only collected for a short period, preventing the tracking of changes over time. Fourth, potential confounding factors that may influence the results were not considered. Finally, all KAP studies are at risk of the social desirability bias, which entails that some participants can be tempted to answer what they know they should think or do instead of what they are really thinking or doing [65, 66].

## Conclusion

In conclusion, patients with dental arch deficiencies have limited knowledge, positive attitudes, and moderate practice toward tooth loss and dentures. These factors may be influenced by various demographic factors such as gender, age, education, employment, household income, marital status, smoking, drinking, disease duration, medical insurance, family history, treatment, and underlying diseases.

### Electronic supplementary material

Below is the link to the electronic supplementary material.

Supplementary Material 1

Supplementary Material 2

Supplementary Material 3

## Data Availability

All data generated or analyzed during this study are included in this article and supplementary information files.

## Abbreviations

KAP: knowledge, attitude, and practice

## References

[1] Helal O, Göstemeyer G, Krois J, Fawzy El Sayed K, Graetz C, Schwendicke F (2019). Predictors for tooth loss in periodontitis patients: systematic review and meta-analysis. J Clin Periodontol.

[2] Kassebaum NJ, Bernabé E, Dahiya M, Bhandari B, Murray CJ, Marcenes W (2014). Global burden of severe tooth loss: a systematic review and Meta-analysis. J Dent Res.

[3] Jordan AR, Stark H, Nitschke I, Micheelis W, Schwendicke F (2021). Epidemiological trends, predictive factors, and projection of tooth loss in Germany 1997–2030: part I. missing teeth in adults and seniors. Clin Oral Investig.

[4] Silva Junior MF, Batista MJ, de Sousa M (2019). Risk factors for tooth loss in adults: a population-based prospective cohort study. PLoS ONE.

[5] Schierz O, Baba K, Fueki K (2021). Functional oral health-related quality of life impact: a systematic review in populations with tooth loss. J Oral Rehabil.

[6] Gupta R, Gupta N, Weber DK. Dental Implants. In StatPearls Treasure Island (FL) ineligible companies. 2024.

[7] Burns DR, Beck DA, Nelson SK (2003). A review of selected dental literature on contemporary provisional fixed prosthodontic treatment: report of the Committee on Research in fixed prosthodontics of the Academy of Fixed Prosthodontics. J Prosthet Dent.

[8] Schierz O, Reissmann D (2016). Influence of guidance concept in complete dentures on oral health related quality of life - canine guidance vs. bilateral balanced occlusion. J Prosthodont Res.

[9] Nagarsekar A, Gaunkar R, Aras M (2016). Knowledge, attitude, and practice of dental professionals regarding the effect and management of food impaction associated with fixed partial denture prostheses: a survey. J Indian Prosthodont Soc.

[10] Palati S, Ramani P, Shrelin HJ, Sukumaran G, Ramasubramanian A, Don KR (2020). Knowledge, attitude and practice survey on the perspective of oral lesions and dental health in geriatric patients residing in old age homes. Indian J Dent Res.

[11] Wong FMF. Factors Associated with Knowledge, attitudes, and practices related to oral care among the Elderly in Hong Kong Community. Int J Environ Res Public Health. 2020;17. 10.3390/ijerph17218088 PMC7672548 33147890

[12] Balwanth S, Singh S (2023). Caregivers’ knowledge, attitudes, and oral health practices at long-term care facilities in KwaZulu-Natal. Health SA.

[13] Modikoe MM, Reid M. Nel RJTJotDAoSADTvdTVvS-A. Oral health-related knowledge, attitudes and practices of adult patients in Mangaung Metropolitan Municipality, South Africa. 2019;74.

[14] Zhu L, Petersen PE, Wang HY, Bian JY, Zhang BX (2005). Oral health knowledge, attitudes and behaviour of adults in China. Int Dent J.

[15] Alhoumaidan A, Mohan MP, Doumani M (2019). The knowledge, attitude and practice of fixed prosthodontics: a survey among Qassim dental practitioners. J Family Med Prim Care.

[16] Lipsky MS, Su S, Crespo CJ, Hung M (2021). Men and oral health: a review of sex and gender differences. Am J Mens Health.

[17] Schulze A, Busse M (2016). Gender differences in Periodontal Status and oral Hygiene of non-diabetic and type 2 Diabetic patients. Open Dent J.

[18] Kumar H, Behura SS, Ramachandra S, Nishat R, Dash KC, Mohiddin G (2017). Oral health knowledge, attitude, and practices among Dental and Medical students in Eastern India - A comparative study. J Int Soc Prev Community Dent.

[19] Vishwanathaiah S (2016). Knowledge, attitudes, and Oral Health Practices of School Children in Davangere. Int J Clin Pediatr Dent.

[20] Redmond CA, Blinkhorn FA, Kay EJ, Davies RM, Worthington HV, Blinkhorn AS (1999). A cluster randomized controlled trial testing the effectiveness of a school-based dental health education program for adolescents. J Public Health Dent.

[21] den Boer JCL, van der Sanden WJM, Bruers JJM (2020). Developments in oral health care in the Netherlands between 1995 and 2018. BMC Oral Health.

[22] Listl S, Galloway J, Mossey PA, Marcenes W (2015). Global Economic Impact of Dental diseases. J Dent Res.

[23] Tsakos G, Sheiham A, Iliffe S, Kharicha K, Harari D, Swift CG (2009). The impact of educational level on oral health-related quality of life in older people in London. Eur J Oral Sci.

[24] Wickholm S, Galanti MR, Söder B, Gilljam H (2003). Cigarette smoking, snuff use and alcohol drinking: coexisting risk behaviours for oral health in young males. Community Dent Oral Epidemiol.

[25] Han GS (2011). Relationship between concentration of oral malodor and smoking, drinking, oral health behavior. J Dent Hyg Sci.

[26] Petersen P PE. The world oral health report 2003: continuous improvement of oral health in the 21st century–the approach of the WHO Global Oral Health Programme. Community Dent Oral Epidemiol. 2003;31(Suppl 1):3–23. 10.1046/j..2003.com122.x 15015736

[27] Shearer DM, Thomson WM, Caspi A, Moffitt TE, Broadbent JM, Poulton R (2012). Family history and oral health: findings from the Dunedin Study. Community Dent Oral Epidemiol.

[28] Botelho J, Machado V, Proença L, Bellini DH, Chambrone L, Alcoforado G (2020). The impact of nonsurgical periodontal treatment on oral health-related quality of life: a systematic review and meta-analysis. Clin Oral Investig.

[29] Svendsen MT, Bak CK, Sorensen K, Pelikan J, Riddersholm SJ, Skals RK (2020). Associations of health literacy with socioeconomic position, health risk behavior, and health status: a large national population-based survey among Danish adults. BMC Public Health.

[30] Yavuz BŞ, Şener M, BEKİROĞLU N, Kargul B (2023). Assessment of Self-reported oral Health attitudes and behaviors of a Group of Dental students using the Hiroshima University-Dental behavioural inventory in Turkey. Eur Ann Dent Sci.

[31] AKTAŞ A, TÜZ H, KARACA Ç. URHAN Ş. Awareness, knowledge, and attitude regarding dental implants in patients attending a University Dental Clinic. Clin Dent Res. 2021;45.

[32] Sanaeinasab H, Saffari M, Taghavi H, Karimi Zarchi A, Rahmati F, Al Zaben F (2022). An educational intervention using the health belief model for improvement of oral health behavior in grade-schoolers: a randomized controlled trial. BMC Oral Health.

[33] Mishra SK, Chowdhary R (2019). Patient’s oral health-related quality of life and satisfaction with implant supported overdentures -a systematic review. J Oral Biol Craniofac Res.

[34] Lopes ABS, Ramos-Jorge ML, Machado GF, Vieira-Andrade RG, Ramos-Jorge J, Fernandes IB (2022). Longitudinal evaluation of determinants of the clinical consequences of untreated dental caries in early childhood. Community Dent Oral Epidemiol.

[35] Josefsson E, Lindsten R (2019). Treatment of missing maxillary lateral incisors: a clinical and aesthetic evaluation. Eur J Orthod.

[36] Schneider UE, Moser L, Pellitteri G, Siciliani G (2018). Orthodontic space closure vs. implant-borne crowns in patients with congenitally missing maxillary lateral incisors. J Clin Orthod.

[37] Emami E, de Souza RF, Kabawat M, Feine JS (2013). The impact of edentulism on oral and general health. Int J Dent.

[38] Folse HJ, Mukherjee J, Sheehan JJ, Ward AJ, Pelkey RL, Dinh TA (2017). Delays in treatment intensification with oral antidiabetic drugs and risk of microvascular and macrovascular events in patients with poor glycaemic control: an individual patient simulation study. Diabetes Obes Metab.

[39] Harrison R (2014). Motivational interviewing (MI) compared to conventional education (CE) has potential to improving oral health behaviors. J Evid Based Dent Pract.

[40] Nagarale R, Todkar M, Jaweed UM, Rajkotwala I, Rao J, Patil S (2022). Assessment of knowledge, attitude and practice regarding replacement of missing teeth among general population in Pune city. Intl J Appl Dent Sci.

[41] Gupta V, Singh S, Singhal P, Gupta P, Gupta B, Kumar S (2022). Perception, awareness, and practice about Missing Teeth, Prosthetic options, and knowledge about Dental implants as a treatment modality in the Adult Population of Jharkhand State: A Hospital-based study. J Pharm Bioallied Sci.

[42] Akeel R (2003). Attitudes of Saudi male patients toward the replacement of teeth. J Prosthet Dent.

[43] Ravikumar V, Siwa Teja SV, George V (2015). A survey to evaluate the awareness of various treatment modalities to replace missing teeth among patients visiting Kasturba Hospital, Manipal: a prosthodontic perspective. IA Original Res.

[44] Poudel P, Griffiths R, Wong VW, Arora A, Flack JR, Khoo CL (2018). Oral health knowledge, attitudes and care practices of people with diabetes: a systematic review. BMC Public Health.

[45] Al-Wesabi AA, Abdelgawad F, Sasahara H, El Motayam K (2019). Oral health knowledge, attitude and behaviour of dental students in a private university. BDJ Open.

[46] Borgan SM, Marhoon ZA, Whitford DL (2013). Beliefs and perceptions toward quitting waterpipe smoking among cafe waterpipe tobacco smokers in Bahrain. Nicotine Tob Res.

[47] Castrejón-Pérez RC, Borges-Yáñez SA, Irigoyen-Camacho ME, Cruz-Hervert LP (2017). Negative impact of oral health conditions on oral health related quality of life of community dwelling elders in Mexico city, a population based study. Geriatr Gerontol Int.

[48] Wang TF, Fang CH, Hsiao KJ, Chou C (2018). Effect of a comprehensive plan for periodontal disease care on oral health-related quality of life in patients with periodontal disease in Taiwan. Med (Baltim).

[49] Tadakamadla SK, Tadakamadla J, Kroon J, Lalloo R, Johnson NW (2020). Effect of family characteristics on periodontal diseases in children and adolescents-A systematic review. Int J Dent Hyg.

[50] Sisson KL (2007). Theoretical explanations for social inequalities in oral health. Community Dent Oral Epidemiol.

[51] Narain KDC, Skrine Jeffers K (2020). Exploring the Relationship between Self-Employment and Health among blacks. Health Equity.

[52] Dzodzomenyo S, Narain KDC (2022). Exploring the relationship between self-employment and women’s cardiovascular health. BMC Womens Health.

[53] Shahnazi H, Ahmadi-Livani M, Pahlavanzadeh B, Rajabi A, Hamrah MS, Charkazi A (2020). Assessing preventive health behaviors from COVID-19: a cross sectional study with health belief model in Golestan Province, Northern of Iran. Infect Dis Poverty.

[54] Modini M, Joyce S, Mykletun A, Christensen H, Bryant RA, Mitchell PB (2016). The mental health benefits of employment: results of a systematic meta-review. Australas Psychiatry.

[55] Gaffar BO, El Tantawi M, Al-Ansari A, AlAgl AS (2016). Association between oral health knowledge and practices of Saudi pregnant women in Dammam, Saudi Arabia. East Mediterr Health J.

[56] Albasry Z, Alhaddad B, Benrashed MA, Al-Ansari A, Nazir MA (2019). A cross-sectional analysis of Dental Care utilization among pregnant women in Saudi Arabia. Open Access Maced J Med Sci.

[57] Okamoto Y, Tsuboi S, Suzuki S, Nakagaki H, Ogura Y, Maeda K (2006). Effects of smoking and drinking habits on the incidence of periodontal disease and tooth loss among Japanese males: a 4-yr longitudinal study. J Periodontal Res.

[58] Umemori S, Aida J, Tsuboya T, Tabuchi T, Tonami KI, Nitta H (2020). Does second-hand smoke associate with tooth loss among older Japanese? JAGES cross-sectional study. Int Dent J.

[59] Finlayson TL, Gansky SA, Shain SG, Weintraub JA (2010). Dental utilization among hispanic adults in agricultural worker families in California’s Central Valley. J Public Health Dent.

[60] Foo P, Sampson W, Roberts R, Jamieson L, David D (2012). General health-related quality of life and oral health impact among australians with cleft compared with population norms; age and gender differences. Cleft Palate Craniofac J.

[61] Reda SF, Reda SM, Thomson WM, Schwendicke F (2018). Inequality in utilization of Dental services: a systematic review and Meta-analysis. Am J Public Health.

[62] Olusile AO, Adeniyi AA, Orebanjo O (2014). Self-rated oral health status, oral health service utilization, and oral hygiene practices among adult nigerians. BMC Oral Health.

[63] Kitagawa M, Kurahashi T, Matsukubo T (2017). Relationship between General Health, Lifestyle, oral health, and Periodontal Disease in adults: a large cross-sectional study in Japan. Bull Tokyo Dent Coll.

[64] Feu D, Miguel JA, Celeste RK, Oliveira BH (2013). Effect of orthodontic treatment on oral health-related quality of life. Angle Orthod.

[65] Bergen N, Labonté R (2020). Everything is perfect, and we have no problems: detecting and limiting Social Desirability Bias in qualitative research. Qual Health Res.

[66] Latkin CA, Edwards C, Davey-Rothwell MA, Tobin KE (2017). The relationship between social desirability bias and self-reports of health, substance use, and social network factors among urban substance users in Baltimore, Maryland. Addict Behav.

