# Supplementary material for: Knowledge, attitudes, and practice related to tooth loss and dentures among patients with dental arch deficiencies
Source: BMC Public Health. 2024 Jul 6;24:1810. doi: 10.1186/s12889-024-19310-2 (PMC11227721; doi:10.1186/s12889-024-19310-2)
Supplement: Supplementary file 1 — Supplementary Material 1 [file 12889_2024_19310_MOESM1_ESM.docx]

**Table S1. Distribution of questions answered for knowledge dimension about tooth loss and denture restoration**

| **Items** | **N (%)** | |
| --- | --- | --- |
|  | **Incorrect** | **Correct** |
| K1. Dental arch deficiency refers to the absence of certain teeth within the dental arch, resulting in a reduced total number of teeth than what is considered normal. | 452 (14.28) | 2714 (85.72) |
| K2. Oral conditions like periodontal disease and dental caries can potentially lead to dental arch deficiencies. | 595 (18.79) | 2571 (81.21) |
| K3. Dental arch deficiencies are not hereditary. | 2437 (76.97) | 729 (23.03) |
| K4. Trauma and post-tumor surgery can lead to dental arch deficiencies. | 672 (21.23) | 2494 (78.77) |
| K5. Dental arch deficiencies can impact a patient's chewing, speech assistance, aesthetics, and may also affect the overall health of the oral and maxillofacial system. | 476 (15.03) | 2690 (84.97) |
| K6. The commonly used methods for restoring dental arch deficiencies include denture restorations, including fixed dental prostheses, removable partial dentures, complete dentures, and implant-supported dentures. | 938 (29.63) | 2228 (70.37) |
| K7. Fixed dental prostheses are suitable for cases with a higher number of missing teeth and in situations where the adjacent teeth and periodontal tissues are relatively healthy. | 3072 (97.03) | 94 (2.97) |
| K8. Removable partial dentures can be categorized based on the material used, such as acrylic dentures, metal dentures, and cobalt-chromium alloy dentures. | 1376 (43.46) | 1790 (56.54) |
| K9. Implant-supported dentures are most often affixed using adhesive or screw-retained methods, providing comfort, ease of use, and effective functionality without the need for removal. | 1271 (40.15) | 1895 (59.85) |
| K10. Implant-supported dentures require a surgical procedure, have a shorter treatment duration, and are relatively cost-effective. | 2379 (75.14) | 787 (24.86) |
| K11. For individuals with dental arch deficiencies, the selection of a suitable restoration method necessitates considering various factors, including the number and location of missing teeth, the degree of alveolar ridge deficiency, the health of remaining teeth, potential changes, overall health status, patient preferences, and economic conditions. | 1034 (32.66) | 2132 (67.34) |
| K12. Existing dental and tissue conditions of individuals with dental arch deficiencies may have other lesions and issues, and it is not always necessary to address them before undergoing dental restoration treatment. | 1627 (51.39) | 1539 (48.61) |
