# Supplementary material for: Knowledge, attitudes, and practice related to tooth loss and dentures among patients with dental arch deficiencies
Source: BMC Public Health. 2024 Jul 6;24:1810. doi: 10.1186/s12889-024-19310-2 (PMC11227721; doi:10.1186/s12889-024-19310-2)
Supplement: Supplementary file 2 — Supplementary Material 2 [file 12889_2024_19310_MOESM2_ESM.docx]

**Table S2. Distribution of questions answered for attitude dimension about tooth loss and denture restoration**

| **Items** | **N (%)** | | | | |
| --- | --- | --- | --- | --- | --- |
|  | **Strongly agree** | **Agree** | **Neutral** | **Disagree** | **Strongly disagree** |
| A1. I believe that missing teeth affect chewing/digestive function. | 1610 (50.85) | 1325 (41.85) | 125 (3.95) | 56 (1.77) | 50 (1.58) |
| A2. I believe that missing teeth affect aesthetics. | 1696 (53.57) | 954 (30.13) | 288 (9.1) | 161 (5.09) | 67 (2.12) |
| A3. I believe that missing teeth affect the alignment of teeth, thereby impacting the health of other teeth. | 1715 (54.17) | 1078 (34.05) | 271 (8.56) | 67 (2.12) | 35 (1.11) |
| A4. I believe that missing teeth can lead to other oral conditions such as periodontal disease and dental caries. | 706 (22.3) | 1241 (39.2) | 888 (28.05) | 298 (9.41) | 33 (1.04) |
| A5. I believe that missing teeth can affect the temporomandibular joint. | 1573 (49.68) | 1261 (39.83) | 157 (4.96) | 133 (4.2) | 42 (1.33) |
| A6. I believe that restoration after missing teeth is necessary. | 1554 (49.08) | 1174 (37.08) | 302 (9.54) | 102 (3.22) | 34 (1.07) |
| A7. The cost of denture restoration is the most significant factor influencing my choice of restoration method and materials. | 815 (25.74) | 1349 (42.61) | 868 (27.42) | 126 (3.98) | 8 (0.25) |
| A8. Suitability and comfort are the two most important factors for me in choosing a denture restoration method and materials. | 999 (31.55) | 1542 (48.7) | 595 (18.79) | 24 (0.76) | 6 (0.19) |
| A9. When it comes to choosing denture restoration methods and materials, I believe that good functionality is the most important factor. | 870 (27.48) | 1498 (47.32) | 706 (22.3) | 87 (2.75) | 5 (0.16) |
