# Supplementary material for: Knowledge, attitudes, and practice related to tooth loss and dentures among patients with dental arch deficiencies
Source: BMC Public Health. 2024 Jul 6;24:1810. doi: 10.1186/s12889-024-19310-2 (PMC11227721; doi:10.1186/s12889-024-19310-2)
Supplement: Supplementary file 3 — Supplementary Material 3 [file 12889_2024_19310_MOESM3_ESM.docx]

**Table S3. Distribution of questions answered for practice attitude dimension about tooth loss and denture restoration**

| **Items** | **N (%)** | | | | |
| --- | --- | --- | --- | --- | --- |
|  | **Always** | **Often** | **Sometimes** | **Rarely** | **Never** |
| P1. I will participate in oral health lectures or events to actively learn about missing teeth and denture restoration. | 227 (7.17) | 400 (12.63) | 1115 (35.22) | 971 (30.67) | 453 (14.31) |
| P2. Whenever necessary, I will undergo denture restoration. | 439 (13.87) | 1070 (33.8) | 1273 (40.21) | 314 (9.92) | 70 (2.21) |
| P3. I will have regular dental check-ups. | 475 (15) | 706 (22.3) | 1711 (54.04) | 236 (7.45) | 38 (1.2) |
| P4. I will have regular teeth cleaning. | 489 (15.45) | 684 (21.6) | 1529 (48.29) | 372 (11.75) | 92 (2.91) |
| P5. I will focus on oral hygiene. | 709 (22.39) | 1261 (39.83) | 901 (28.46) | 253 (7.99) | 42 (1.33) |
| P6. If suitable and comfortable, even if the denture restoration method and materials are expensive, I would choose them. | 424 (13.39) | 1154 (36.45) | 996 (31.46) | 545 (17.21) | 47 (1.48) |
| P7. I will follow medical advice and cooperate with all pre-treatment checks and treatments before denture restoration. | 880 (27.8) | 1509 (47.66) | 718 (22.68) | 45 (1.42) | 14 (0.44) |
| P8. I will not use denture restoration to treat missing teeth. | 201 (6.35) | 140 (4.42) | 892 (28.17) | 1446 (45.67) | 487 (15.38) |
